# African American female with renal failure presenting with skin lesions: a case report

**DOI:** 10.4076/1757-1626-2-8701

**Published:** 2009-07-29

**Authors:** Sajitha MF Rahman, Nejla Israel, Tsveti Markova

**Affiliations:** Department of Family Medicine and Public Health Sciences, Wayne State University1100, W. University Drive, Rochester Hills, Michigan 48307USA

## Abstract

Calciphylaxis is a lethal disorder seen in patients with end-stage renal disease and is characterized by painful necrotic skin lesions. The pathophysiology is still unknown. Elevated calcium, phosphorous and parathormone appear to play a role in causing small and medium sized vasculopathy. Diagnosis is delayed, prognosis is poor and mortality remains high. In this article we describe the case of calciphylaxis in a patient with chronic renal failure and multiple medical comorbidities, and discuss diagnostic management, prognosis and treatment options.

## Introduction

Calciphylaxis is rare disfiguring skin condition that is most often associated with end-stage renal disease and long-term dialysis. Calciphylaxis-related morbidity and mortality are significant. The case demonstrates the diagnostic and therapeutic dilemmas associated with management of such patients. The complexity of the patient’s history and multiple medical comorbidities, attempted surgical interventions and inconclusive biopsy results complicates diagnostic process. Close scrutiny of multiple physical assessment findings, historical factors and test results were required for correct diagnosis, assessment of prognosis and treatment options.

## Case presentation

A 78-year-old African American female was admitted with multiple painful ulcers on the lower extremities and possible septicemia. Her medical history included coronary artery disease, peripheral vascular disease, end-stage renal disease on hemodialysis for 7 months, insulin-dependent diabetes, hypertension, hypothyroidism, chronic obstructive pulmonary disease and asthma. Her surgical history included thyroidectomy and left upper extremity graft in October 2005 which failed to maturate and patient did not follow up. Patient was admitted in March 2006 for symptoms of worsening of lower extremity swelling and paroxysmal nocturnal dyspnea when her laboratory results showed BUN-141 mg/dl and creatinine-8.1 mg/dl. Patient underwent fistulogram which showed multiple high-grade stenosis. She had internal jugular catheter placed at the time and was started on daily hemodialysis. When she was discharged 6 days later, her BUN was 33 mg/dl and creatinine was 5.4 mg/dl. She was advised to follow with the dialysis center three times weekly, however patient remained noncompliant with intermittent dialysis. Since the September 2006 hospitalization as described on the case she was dialyzed regularly on Tuesday, Thursday & Saturday. She also had right lower extremity angiogram with an attempted right superficial femoral arterectomy and angioplasty which failed because of severely calcified vessels and multiple wound debridements in the past 3-4 months. Drug therapy included aspirin 81 mg daily, pentoxifylline 400 mg 3 times daily, lisinopril 40 mg daily, amlodipine 10 mg and minoxidil 2.5 mg daily, insulin 70/30 22 units every morning and 12 units every evening, atorvastatin 40 mg daily, albuterol and atrovent inhalers as needed, levothyroxine 150 mcg daily, PhosLo 2 caps 3 times daily, cinacalcet 30 mg daily, colace 100 mg twice daily, hydrocodone 500 mg as needed. Patient denied any exposure to tobacco, ethanol, and intravenous drug abuse. She had 2 children and lived with her son and daughter-in-law who are the primary caretakers. Family history included chronic kidney disease, hypertension, coronary artery disease and diabetes.

**Figure 1.**
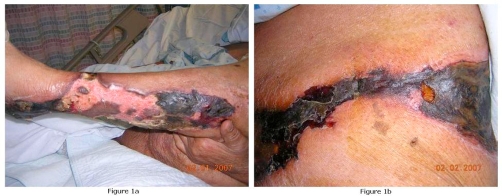


Six months prior to presentation, she was found to have significant hyperparathyroidism (serum intact parathormone of 1133 pg/ml [normal, 10-65]) and hypercalcemia of 11.1 mg/dl [normal, 8.5-10.5]. Meanwhile, she developed 7 × 4 cm ulcer on the posterior aspect of right calf after a burn which remained non-healing for more than 2 weeks. She complained of severe local pain and continued developing multiple ulcerations on both lower extremities. Wound care and pain management was attempted as outpatient. Admission followed due to worsening of ulcerations and poor pain control. On admission, physical examination revealed obese (Ht-154 cm, Wt-84 kgs, BMI-35) African American female in distress due to pain with marked abdominal adiposity, absent pedal pulses and extensive lesions on the hips, buttocks, and proximal thighs with surrounding erythema, induration and dark eschar (Figure 1a & 1b). No other pertinent physical findings were present. Laboratory studies showed serum calcium of 8.6 mg/dl (normal, 8.5-10.5), serum phosphorous of 3.7 mg/dl (normal, 2.5-5.0), calcium-phosphorous product - 31.8, parathormone of 155 pg/ml (normal, 10-65), urea of 35 mg/dl (normal, 7-20) and creatinine of 4.3 mg/dl (normal, 0.8-1.3), alkaline phosphatase-145 IU (normal, 70-230 IU), and serum albumin-1.9 g/dl (normal, 3.5-5.5 g/dl).

X-ray of lower extremities revealed extensive arterial calcifications within the femoral, popliteal artery and smaller branches. Nuclear medicine scan of parathyroid showed adenoma of the right lobe. With significant multiple co morbidities of diabetes, peripheral vascular disease and end stage renal disease, we considered the possibilities of ischemic ulcers and superimposed infection. The patient underwent several investigations, including a lower extremity angiogram with an attempted right superficial femoral arterectomy and angioplasty which failed because of severely calcified vessels and multiple wound debridements in the past 3-4 months. Hypercoagulable state with cryoglobulinemia was also ruled out, with the results of protein C, protein S and antithrombin-3 being in the normal range.

**Figure 2.**
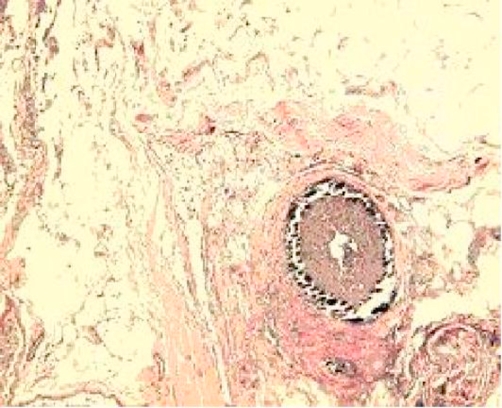


A biopsy of the lower extremity ulcer demonstrated circumferential vascular mural calcification affecting small and medium sized blood vessels with degeneration of elastic fibers and absence of inflammation (Figure 2). During her hospitalization, despite attempts to control deterioration (wound care, pain control, hemodialysis and parenteral antibiotics), leg wounds and general condition worsened over the next 3-4 weeks. She exhibited periodic disorientation. Upon multiple discussions with the family on different treatment options and overall poor prognosis, the patient’s family opted for comfort care under Hospice. Patient expired two days later.

## Discussion

Calciphylaxis is an abnormal skin calcification found mostly in end-stage renal disease patients who have secondary hyperparathyroidism. The integumentary deterioration is related to calcification of soft tissues and blood vessel walls, causing cessation of blood supply. The process of calciphylaxis was originally described by Hans Selye in 1962 following experimental observations in animal models [1]. As the process was considered to be one of ‘induced hypersensitivity’, resulting in local calcification following the two-step process of sensitizing and challenging (analogous to anaphylaxis), it was termed calciphylaxis [2].

An estimated 1% of end-stage renal disease patients develop calciphylaxis yearly, with a potential total prevalence up to 4% of all end-stage renal disease patients [3]. Risk factors include renal impairment, dialysis dependency, females, obesity, warfarin use, hypercoagulable states, diabetes mellitus, atherosclerosis, hypertension, protein malnutrition and those receiving calcium salts and vitamin D therapy. Calciphylaxis occurrence or severity is not predicted on the type or duration of chronic renal failure, the severity of hyperparathyroidism, or the degree of parathyroid hormone elevation.

The pathogenesis of this disease remains elusive [4]. Hypercalcemia, hyperphosphatemia, and hyperparathyroidism are frequently seen with calciphylaxis, but their absence does not exclude the diagnosis. In patients with end stage renal disease, positive calcium balance and use of oral phosphate binders to combat osteodystrophy accelerates uremic vasculopathy in the dialysis population [5]. Exposure of vascular smooth muscle to uremic serum or phosphorus in vitro increases osteopontin expression causing the transformation of vascular smooth muscle cells to an osteoblastic phenotype in the mechanism of medial calcinosis. Endovascular fibrosis is the major cause of ischemia while other proposed hypothesis include luminal obliteration by calcium salts, thrombosis due to acquired protein C deficiency, vasoconstrictor effect of calcium and autonomic dysfunction. Role of protein C deficiency in the pathogenesis of systemic calciphylaxis is still unclear [4].

Microvascular calcification in the intima of small arteries and, to a lesser extent, in the media is noted histologically. The lumen of the vessels is thereby narrowed, and arterial thrombosis is occasionally seen with and without signs of recanalization. Complete vascular occlusion is rarely noted. In consort with the distinctive mural calcification is a superimposed intimal fibroblastic hyperplasia with the presence of giant cells that appears to be the primary cause of the ischemia [6]. This histologic feature in the small arteries with a diameter of approximately 0.04 to 0.1 mm in uremics is almost pathognomonic for calciphylaxis and can be easily differentiated from atheromatosis and peripheral arterial occlusive disease.

The time period for the onset of symptoms of calciphylaxis ranged from less than 1 month to as long as 12 years after the onset of end-stage renal disease (median, 2 yr 9 months) [4]. The classical presentation starts with painful purplish (violaceous) mottled skin lesions that may become plaque like or nodular. They often progress to non-healing ulcers with wound infection and eschar formation, and usually develop gangrene. Involvement of the lower extremities is almost universal. Preserved peripheral pulses favor the diagnosis of calciphylaxis.

The diagnosis of calciphylaxis is based on clinical, biochemical and histopathological features. Laboratory values of calcium, phosphorous, calcium-phosphorous product, parathormone, alkaline phosphatase, urea and creatinine are likely to be significantly elevated. Radiography may reveal subperiosteal bone resorption and ‘pipe-stem’ pattern of vascular calcification. Bone scan may reveal increased tracer accumulation in subcutaneous tissue in a patient with end-stage renal disease and calciphylaxis [7]. Tissue biopsy usually shows multiple discrete foci of calcification within the adipose lobules, in subcutaneous septa, and in the tunica media layer of small-to medium-sized blood vessels. Factors associated with poor prognosis include cutaneous ulcers, extensive tissue involvement, previous renal transplant, proximal lesions, a history of trauma and higher preoperative leukocyte counts (over 20,000).

Differential diagnosis for similar ulcerations include vasculitis, systemic lupus erythematosus, cryoglobulinemia, scleroderma, disseminated intravascular coagulation, cholesterol emboli, Henoch-Schonlein purpura, homocysteinemia, heparin-associated gangrene, warfarin-induced skin necrosis and bacterial endocarditis. Investigations including vasculitis screen, estimation of cryoglobulins and cryofibrinogens, fibrin degradation products, antiphospholipid antibodies and Doppler assessment of limb vessels need to be considered [8].

Diligent wound care, avoidance of trauma, and appropriate antibiotic usage together with nutritional support and adequate pain control are important aspects of general care of these patients. Wound care includes gentle handling and moist wound dressing with material such as petrolatum-impregnated gauze to minimize tissue damage and local debridement. Sterile maggot therapy, pentoxyfillin and hyperbaric oxygen is used to treat ulcerated areas [9].

Medical management involves normalization of altered calcium and phosphorous levels. Food high in calcium and phosphorous must be limited. Calcium and aluminum-free phosphate binders such as renagel have been found to be useful in the management of renal osteodystrophy. Laboratory values have to be monitored so the calcium-phosphorous product, parathormone level, total protein and pre-albumin level stay within normal limits. Increased frequency of haemodialysis too has been employed as a management strategy.

Good pain relief is the most important and challenging aspect of symptom control [10]. Severe pain, opioid resistance, and the susceptibility of these patients to opioid toxicity mandate a broad approach to analgesia. Combination of an opioid, benzodiazepine, and ketamine appears to give the best analgesia. Other options include inhaled nitrous oxide, and potent parenteral anti-inflammatory agents such as ketorolac. The use of spinal analgesia with local anesthetic agents has also been used with limited effectiveness. Fentanyl analogs are a good choice in these patients because of their hepatic clearance, stability during dialysis, and lack of clinically active metabolite accumulation.

Surgical treatment is only variably successful. Total parathyroidectomy or parathyroidectomy with auto transplantation is recommended for patients with hyperparathyroidism [10]. Severe necrosis of extremities often involves amputation.

Early involvement of a multidisciplinary palliative care team is beneficial. Given the poor prognosis of calciphylaxis, the team also provides a bridge to terminal care as the patient’s condition deteriorates [10].

## Conclusion

Calciphylaxis is a potentially lethal syndrome seen in patients with end-stage renal disease and secondary hyperparathyroidism. It may, however, be associated with other disease entities in the absence of renal or parathyroid disease. A high index of suspicion, early intervention, and an active multidisciplinary medical and surgical approach are vital aspects of the management strategy.
